# Behavioural susceptibility to environmental influences in obesity– evidence from a companion animal model

**DOI:** 10.1186/s12917-025-04990-8

**Published:** 2026-01-05

**Authors:** Anna Morros-Nuevo, Carina Salt, Jessica Pavey, Jodie F. Wainwright, Marie Dittmann, Benjamin Keep, Natalie Jessica Wallis, Eleanor Raffan

**Affiliations:** 1 Department of Physiology, Development and Neuroscience, University of Cambridge, Cambridge, Cambridgeshire UK; 2 Waltham Petcare Science Institute, Leicestershire, UK; 3 School of Biological and Behavioural Sciences, Queen Mary University of London, London, UK

**Keywords:** Obesity, Dog, Canine, Food motivation, Appetite, Overweight, Eating behaviour

## Abstract

Obesity is often pejoratively viewed as the consequence of poor self-restraint with the influence of genetics on individuals’ drive to eat overlooked. We studied pet dogs (*Canis familiaris*) as a compelling animal model in which obesity develops spontaneously, subject to similar environmental influences as in their human counterparts and in which artificial selection means dogs within a breed are genetically homogeneous. In electronic health records from 1.1 million dogs, we showed wide variation in the probability of obesity in different breeds, evidence that obesity is highly heritable in this species. Using a validated questionnaire in ~ 15,000 dog/owner dyads we show that food motivation is a key driver of obesity in dogs, and that high food motivation renders affected dogs particularly susceptible to an obesogenic environment. As well as being of veterinary interest, this is of relevance to human obesity as compelling, data-driven evidence of how behavioural susceptibility to environmental risk governs obesity outcome.

**Supplementary Information:**

The online version contains supplementary material available at 10.1186/s12917-025-04990-8.

## Background 

Despite high heritability [67, 71, 75], human obesity is often pejoratively viewed as the consequence of poor self-regulation with the influence of genetics on individuals’ hunger, satiety and complex eating behaviours frequently overlooked [13, 21, 46, 63, 77]. The behavioural susceptibility theory posits that genetically driven variation in human appetite and eating behaviour causes variation in individual’s susceptibility to an ‘obesogenic’ environment. Available evidence supports the theory but is somewhat limited by the difficulty of quantifying eating behaviour and environmental exposure to obesity risk factors at scale in large numbers of individuals [3, 37].

A canine obesity epidemic mirrors that observed in humans [4, 6, 12, 26, 36, 39, 41, 66, 70] and is also often blamed on a lack of control of diet and exercise from their human owners [10, 32, 65]. Evidence that obesity is highly heritable in dogs (*Canis familiaris*) comes from strong breed predispositions and work that shows the effect of particular mutations on obesity risk [8, 58, 59, 73]. Selective breeding for specific traits means that, whilst there is great diversity across the species, pedigree dogs of the same breed are genetically very similar and tend to share similar risks of developing even complex genetic conditions [9, 11, 40, 73].

In this study we exploited these unique features of the canine model and quantified both dogs’ eating behaviour and their environmental exposure to food and activity in large populations, including electronic health records from > 1 million purebred dogs and ‘Dog obesity risk assessment’ (DORA) questionnaire [59] responses from a further 14,960 dog/owner dyads. This previously validated questionnaire is an owner-reported measure of food-motivation (a combined measure of dogs’ food responsiveness and satiety, lack of selectivity, and interest in food) and owner control of diet and activity levels.

This work uses dogs as a model of how genetic variation can influence eating behaviour, and how, in turn, variation in eating behaviour renders some individuals particularly susceptible to developing obesity if exposed to environmental factors which predispose them to weight gain [24, 42, 60].

## Results

### Food motivation is highly variable between individuals and across breeds

We previously validated the DORA questionnaire [59], an owner-reported measure of eating behaviour in the home environment. The questionnaire measures dogs’ food motivation using owners’ responses on a Likert scale to 13 statements about their dog such as ‘my dog gets excited when there is food around’, ‘my dog takes his/her time eating a meal’ and ‘my dog hangs around when I am preparing or eating human food’. We analysed data from 14,960 respondents to the DORA questionnaire, for which food motivation score (FMS) ranged from 0 to 1 with mean 0.633 and SD 0.246.

**Fig. 1:**
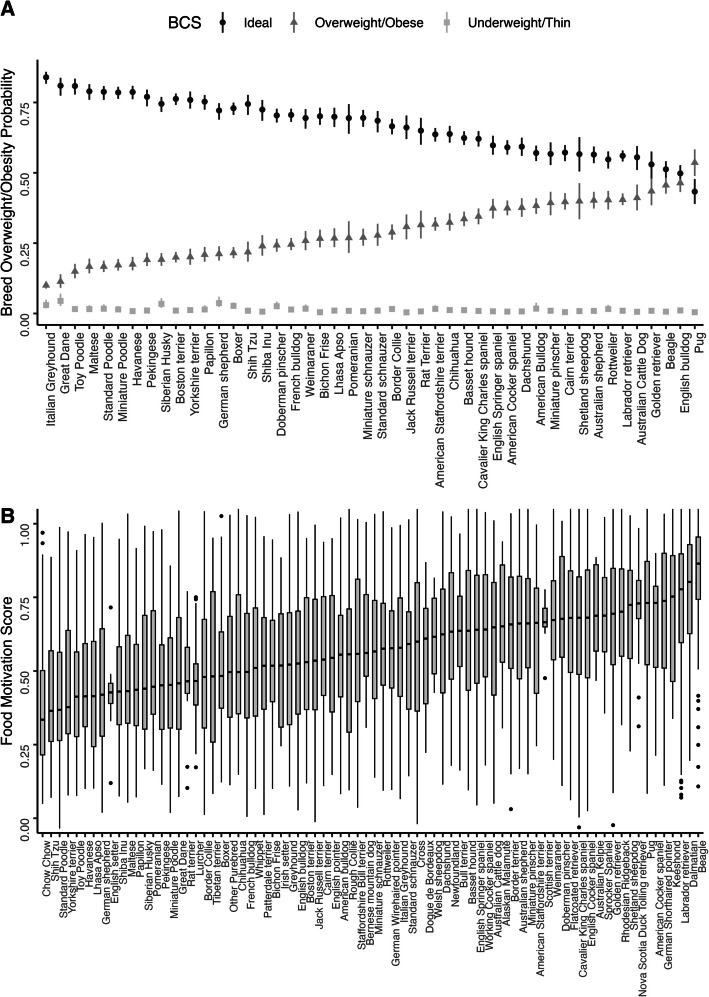
Food motivation and overweight/obesity probability are highly variable across breeds and are highly correlated. **A** Predicted average probabilities (0–1) and 95% confidence intervals of being underweight (BCS 1–2/5), ideal weight (3/5) or overweight/obese (4–5/5), by breed, ordered by the ascending probability of being overweight. These were predicted from electronic medical records of 1.1 million neutered dogs of 46 different breeds seen at Banfield Pet Hospitals between March 4th 2015 and Jan 31 st 2018. **B** Box and whisker plot is shown for breed average Food Motivation Score (0–1) from 14,960 responses to the previously validated DORA questionnaire [59], calculated with weighted effect of sex, age and neuter status for each breed. Graph shows median (horizontal black line), interquartile range (grey area), upper and lower extremes (vertical black line) and outliers (dots).

Food motivation was significantly different across dog breeds (Fig. 1B). Linear regression models were compared using Akaike Information Criterion, and we found that sex, age and neutering status, which were previously identified as influencing Food Motivation Score in dogs [59], accounted for only 2.6% of variability (R^2^ = 0.0256, *p* < 0.001). Remarkably, this compared to 16% when breed was added into the model (R^2^ = 0.1643, *p* < 0.001). Since dogs within a breed are genetically homogeneous but between breeds there is genetic diversity, this strongly suggests that food motivation is a highly heritable trait in dogs, as in other species [29, 51, 64].

### Breed obesity risk is highly variable

Electronic health records for 1,149,591 neutered (gonadectomized) purebred dogs of 46 different breeds, aged between 1 and 15 years old, that visited Banfield Pet Hospitals (a primary care veterinary hospital network in the United States) between March 4th 2015 and Jan 31 st 2018, were used to extract pet signalment data and Body Condition Score (BCS), a measure of adiposity using a combination of visual and haptic cues [18, 19, 34, 45]. The breeds were selected to be those also present in the Food Motivation Score data. Body Condition Score was treated as a 3-category variable, according to the available medical records, with possible values being underweight (1–2/5), ideal weight (3/5) and overweight/obese (4–5/5) [18, 19, 45]. We modelled the probability of each of these categories by breed, taking into account age and sex, and showed there was a large variation in the proportion of overweight and obese dogs between breeds (Fig. 1A).

High risk breeds identified (e.g. Labrador Retriever, Pug and Beagle) are similar to those reported in other smaller studies [39, 55, 69, 70] but the scale of our dataset provides evidence about many other breeds. Although some of the variability observed between breeds may be down to dogs’ lifestyle or owners’ perceptions of what body shape is appropriate for the breed [2, 31, 32, 54, 68], this clear breed variation strongly supports that obesity risk is in large part mediated by genetics in dogs, as in other species [29, 51, 64].

### Food motivation explains much of the variation in individual dog obesity

To assess obesity in dogs with Food Motivation Scores, we also collected information on these dogs’ Bo al and every point increase above that is associated with approximately dy Condition Scores (BCS) on a 9-point [45]. A BCS of 4–5 is considered optim 8% increase in body fat mass [18, 19, 34, 45].

**Fig. 2:**
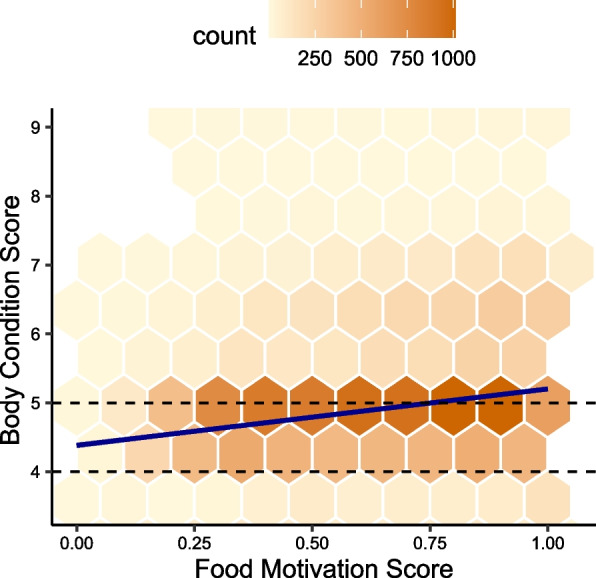
Food motivation is strongly associated with body condition score in dogs. This figure shows a Hexplot to demonstrate distribution of BCS and FMS at an individual level. The dashed lines at BCS 4 and 5 indicate optimal BCS range. The regression line is overlain in blue

Body Condition Score was significantly positively correlated with Food Motivation Score in individual level data (*r* = *0.19*, *p* < *0.001)* (Fig. 2). When a regression model with two-way interactions to predict BCS included only conventional risk factors (BCS ~ Sex*NeuterStatus + NeuterStatus*Age + Sex:Age), the variation explained by the model was 4.7% (R^2^ = 0.047, *p* < 0.001). Adding Food Motivation Score into the model increased the variability explained to 6.8% (R^2^ = 0.067, *p* < 0.001). While age, sex and neuter status have largely been considered the main risk factors for canine obesity, this data shows that food motivation has a similar impact on individual obesity risk.

Breed and food motivation were strongly associated (Fig. 1B) so it was inappropriate to include both in a regression model. However, adding breed to the model instead of Food Motivation Score increased variability explained from 4.7% to 10% (R^2^ = 0.102, *p* < 0.001). Since breed is a surrogate marker of genetic similarity, this indicates that obesity is heritable in dogs, as in other species, and that genetically driven variation that was not captured with the DORA questionnaire (e.g. other eating behaviours or alternative physiological or metabolic mechanisms) are also impacting canine obesity.

Previous reports have shown owners tend to score their dogs as having BCS closer to ideal than reality [31, 31, 32, 54, 62, 68, 78]. In a subset of 618 British dogs with both veterinary assigned BCS and owner-reported BCS we confirmed this was true, as expected (Additional file 1) and the measures were moderately correlated (*r* = 0.49, *p* < 0.001). This is likely to lead to underestimation of the effect sizes reported in our study. Despite this, the moderate and highly significant correlation means the directionality and comparative size of the effects reported are robust.

### Breed obesity risk is in large part explained by breed variation in food motivation

**Fig. 3:**
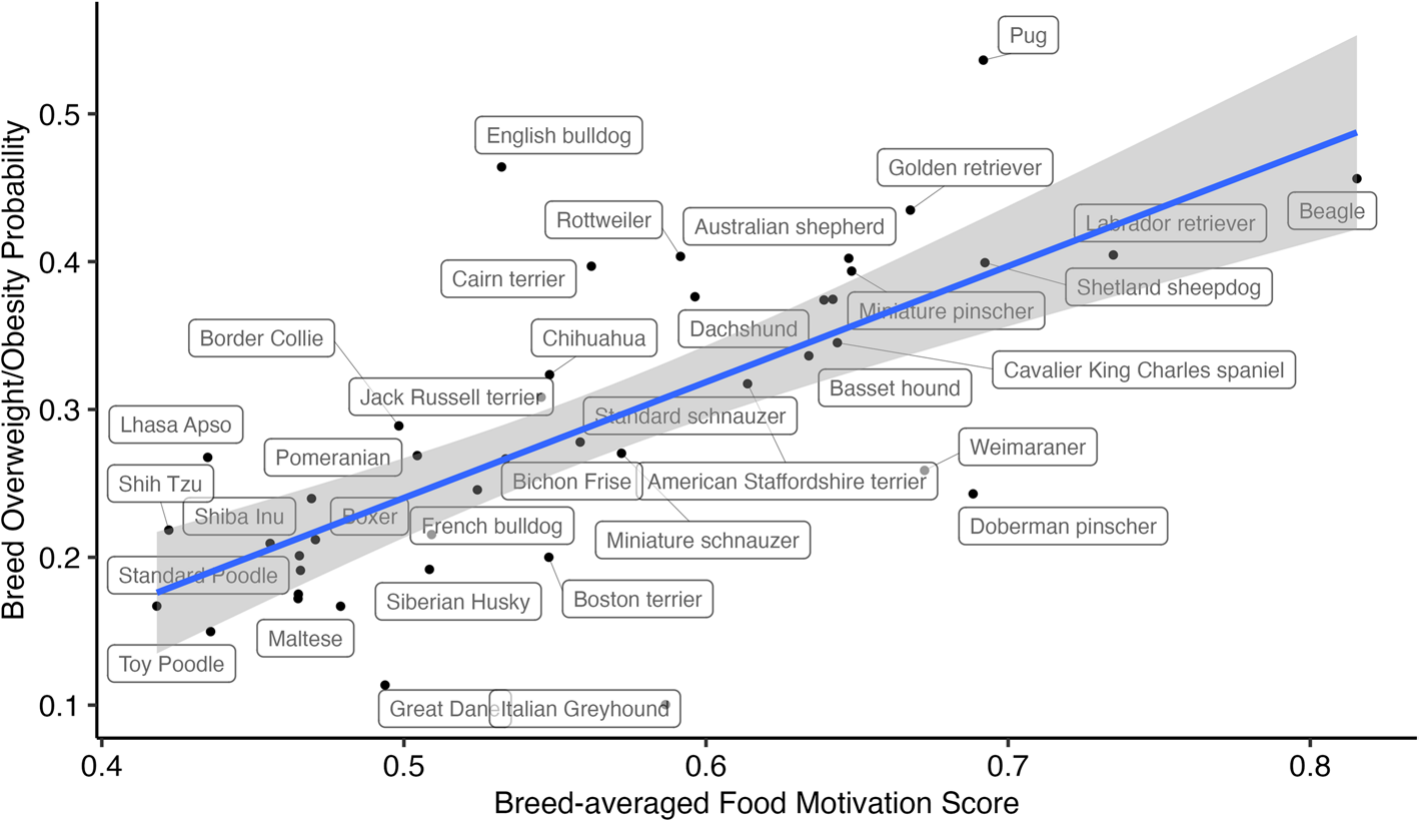
Breed-averaged food motivation and breed-averaged probability of obesity/overweight are highly correlated. Scatterplot with regression line of breed average overweight/obesity probability and breed averaged food motivation (R^2^ = 0.51, *p* < 0.001) for the 46 breeds present in both datasets, where labels show the breed corresponding to each dot, line of best fit is shown in blue and grey shade shows 95% confidence interval.

We modelled breed average Food Motivation Score from 14,482 dogs collected predominantly in the UK accounting for age, sex and neuter status (Fig. 1B) and assessed it against breed obesity probability obtained from the medical records of ~ 1.1 m US dogs (Fig. 3). In this breed average data here was a strong positive correlation between the two variables (*r* = 0.711, *p* < 0.001) and over half the variability in predicted breed overweight/obesity probability was explained by differences in breed average food motivation alone (R^2^ = 0.5053, *p* < 0.001). These data support our hypothesis that breed variation in obesity risk is in large part mediated by variability in food motivation.

### Owners try, but often fail, to control weight in highly food motivated dogs

The DORA questionnaire also quantifies whether owners give dogs human food (‘Restriction of Human Food Score’), how much exercise dogs get (‘Exercise Score’) and strategies to control dogs weight (‘Owner Intervention Score’) which captures interventions such as weighing food or changing the diet to promote a healthy weight. The weighted average of these three factors was also obtained (‘Owner Control Score’). This is, in effect, a measure of the degree to which dogs are exposed to obesogenic factors in their home environment, since the items report on management behaviours which have previously been shown to be environmental risk factors for obesity [59].

The positive association between Food Motivation Score and BCS reported above was present despite food motivation also being positively correlated with ‘Owner Control’ (*r* = 0.12, *p* < 0.001). This suggested that owners of more highly food motivated dogs report exerting greater control over their dogs’ weight but, despite that, highly food motivated dogs had higher adiposity in the population.

To understand that better, we scrutinised the components of the overall score. Food Motivation Score was significantly positively correlated with ‘Owner Intervention’ (*r* = 0.29, *p* < 0.001) but negatively correlated with ‘Restriction of Human Food’ (*r* = −0.07, *p* < 0.001) and there was no significant correlation with ‘Exercise’ (Additional file 2). These findings suggest that while owners recognise their pets’ high food motivation and put measures in place to restrict their food intake, such as measuring food portions, they are more likely to give human food to highly food motivated dogs, despite this being widely recognised as suboptimal nutrition and a risk factor for weight gain [5, 17, 20, 61, 66].

We considered whether owners tend to provide different amounts of exercise to dogs of different breeds or offer different amounts of food and whether this could explain the breed differences in obesity probability (rather than food motivation). However, breed-averaged Exercise explained only 1.1% of variability in breed-obesity probability (R^2^ = 0.011, *p* = 0.497) and had no significant correlation with it (*r* = 0.1, *p* = 0.496). Breed-averaged Owner Control explained more of the breed overweight/obesity probability (13% of variability, R^2^ = 0.129, *p* = 0.017) and had a mild but significant positive correlation (*r* = 0.359, *p* = 0.017), suggesting that control tends to be greater in breeds with higher obesity probability, as in the individual-level data. These results support that differences in obesity prevalence or risk between breed are primarily due to underlying food motivation rather than differing management of the diet and exercise environment by owners (Additional file 3).

### Exposure to environmental risk factors has greater impact in highly food motivated dogs

Although across the population food motivation was associated with higher BCS, there are many highly food motivated dogs with healthy body weight. To test how dogs’ biologically determined obesity risk interacts with environmental risk factors we used Food Motivation Score as a marker of underlying risk and stratified the population of dogs with individual level data on food motivation and obesity as high, medium or low obesity risk according to Food Motivation Score tertile.

**Fig. 4:**
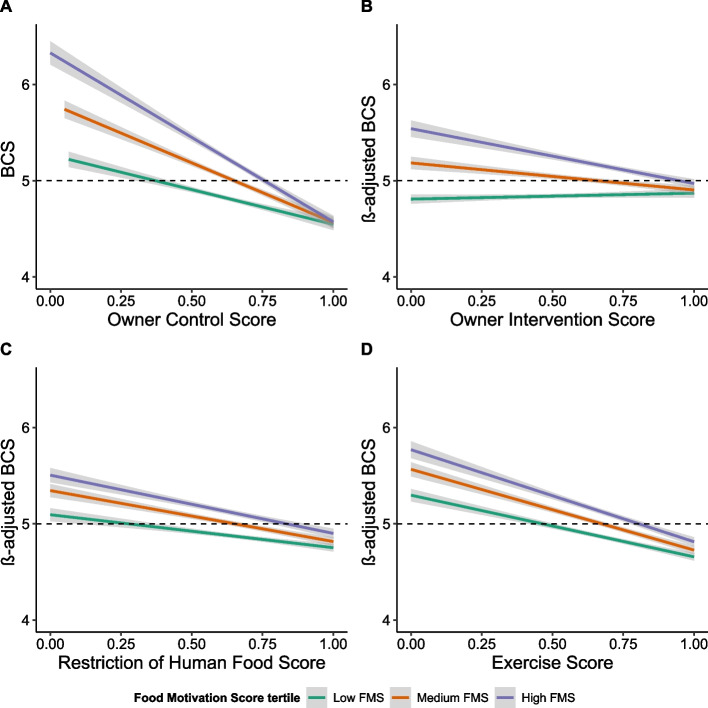
High- risk dogs are more susceptible to environmental factors related to exercise and food availability (Additional file 4). Regression Plot of BCS against Owner Control Score (0–1) (**A**), and Partial Regression Plots of β-adjusted BCS against Owner Intervention Score (**B**), Restriction of Human Food Score (**C**) and Exercise Score (**D**), by Food Motivation Score tertiles. Line of best fit is shown for high, medium and low tertile food motivation subgroup with 95% confidence intervals represented by the shaded grey area. Black dashed line represents optimal adiposity (BCS = 5). β-adjusted BCS was calculated with the effect sizes β of all variables and interaction terms contributing to explain BCS variability after obtaining minimal model with AIC.

Overall ‘Owner Control’ was negatively correlated with BCS (*r* = −0.12, *p* < 0.001) (Fig. 4) and owners who exerted high control over their dogs’ weight could successfully maintain them at a healthy BCS irrespective of risk group. However, for a given degree of ‘Owner Control’, BCS (mean ± sd) was significantly higher (t(8430) = −17.3, adj. *p* < 0.001) for dogs in the high obesity risk group (highest ‘Food Motivation Score’ tertile) compared to dogs in the lowest obesity risk group (5.24 ± 1 vs 4.85 ± 0.79, respectively).

We modelled the effect size (*β*) on BCS of obesity risk group, ‘Owner Control’ subfactors and other known risk factors (age, sex and neuter status) using linear regression. The minimal model was obtained through stepwise elimination process based on Akaike Information Criterion, which is shown below (in which asterisks indicate retention of standalone variables and two-way interaction):

$$\begin{aligned} \text{Body Condition Score }\sim &\text{ Owner Intervention}*\text{Food Motivation tertile }\\&+\text{ Restriction of Human Food}*\text{Food Motivation tertile }\\&+\text{ Exercise}*\text{Food Motivation tertile }+\text{ Sex}*\text{Food Motivation tertile }\\&+\text{ Neuter Status}*\text{Food Motivation tertile }+\text{ Sex}*\text{Neuter Status }+\text{ Age} \end{aligned}$$

The model explains 14% of variability in BCS across the population (R^2^ = 0.136, *p* < 0.001) and quantifies the effect size and significance of each risk factor in the contrasting risk groups (Additional file 4). The largest effect size was attributed to ‘Food Motivation’ group, corroborating findings from non-grouped data presented above (Fig. 2) and show that food motivation is a driver of adiposity in dogs. Previously known risk factors (age, sex and neuter status) also remained in the model but had smaller effects than ‘Owner Control’ subfactors and ‘Food Motivation’. Importantly, the effect size of each component of ‘Owner Control’ was greater in the most highly food motivated dogs, less in the medium risk group and lowest in the lowest obesity risk group.

## Discussion

These data reveal how dogs with high food motivation are particularly susceptible to an obesogenic environment (low owner control) compared to those with low food motivation. Highly food motivated individuals are likely to develop severe obesity given ready access to food and little need to exercise, in contrast to those with low food motivation who are relatively protected against obesity even where the environment might promote it. Alternatively, it takes greater effort to maintain normal body weight in dogs with higher inherent obesity risk and food motivation. Although common sense, this is a compelling and data-driven illustration of how inherent obesity risk alters susceptibility to environmental risk factors concerning food availability and physical activity.

Notably, in medium and high-risk groups, greater ‘Owner Intervention’ was associated with lower BCS, but in the least food motivated dogs, this effect was reversed. We interpret this surprising result as owners of ‘fussy’ dogs not needing to intervene to prevent weight gain but, in some cases, intervening to promote food intake in their pets to meet a perceived normal weight greater than that determined by the dog’s homeostatic drives.

There was a strong negative relationship between ‘Exercise Score’ and BCS, consistent with that reported by others [7, 59, 76]. However, we acknowledge that causality cannot be inferred, and the association may exist in part because overweight dogs are less keen to exercise or unable to do so due to obesity co-morbidities [1, 33, 35, 38, 39, 43, 50]. Importantly, while at an individual level higher exercise is correlated with lower adiposity, exercise level does not explain breed differences in overweight/obesity. Notably, whereas ‘Restriction of Human Food’ and ‘Owner Intervention’ were different between high and low risk groups, there was no difference in mean ‘Exercise Score’ despite high-risk dogs being more overweight (Additional file 5). This suggests owners do not alter their pets’ exercise to moderate the impact of their high food drive on body weight, which is further supported by the lack of association between exercise and obesity phenotype at a breed level.

Although restricting human food was associated with lower BCS across all groups, the mean score for ‘Restriction of Human Food Score’ was significantly lower in highly food motivated dogs (Additional file 5). Highly food motivated dogs have greater food responsiveness, are less discriminating about which foods they eat and are more interested in food, traits that manifest as attention seeking at mealtimes or hanging around when food is being prepared, which owners commonly interpret as ‘begging for food’ [59]. The data therefore supports evidence from the canine behaviour literature that dogs can effectively communicate their desire for food [22, 23, 30, 44, 47, 52] and reinforce titbit feeding by owners with behaviours that are perceived as ‘gratitude’ or which strengthen the pet-owner bond [27, 48, 53, 72]. That is, owners may have good intentions to manage their pets’ weight (with ‘owner interventions’ and ‘exercise’) but tend to succumb to canine ‘pester power’ [15, 16, 53].

For the breed-average comparisons, we considered whether studying obesity data from American dogs alongside food motivation data from geographically diverse (but predominantly British) dogs might have affected our results, as discrepancies may exist between genetic risk for high appetite/obesity or in ownership caregiving styles across the populations. Similarly, the breed average obesity results were estimated in neutered dogs only but were compared to breed average food motivation scores calculated from both neutered and entire dogs (which were corrected for comparison by the effect size of neutering in the modelling). These theoretical or actual differences might be expected to cause a type II error (leading to a false negative result or reduced strength of association) but there is no reason to believe they would have introduced a type I error. Consequently, although the true association may actually be stronger that that we reported, we are confident the associations reported are valid.

### Practical implications for canine healthcare

We have demonstrated that canine obesity is in large part mediated by variation in food motivation both in individuals and across breeds. The clear breed differences in food motivation and obesity prevalence strongly suggest that both are heritable traits in dogs. In practical terms, the data shows owners can successfully keep even the most highly food motivated dogs slim. However, they need to exert much greater effort to maintain highly food motivated dogs at a healthy weight while even lax management of a dog with very low food motivation is unlikely to lead to them becoming overweight. In the contrary, whilst exercise level might be relevant at an individual basis, it is not impactful at a population level.

This has important implications for preventative healthcare and individual weight management in pet dogs: we would recommend that prevention efforts are targeted at the most food motivated and high-risk dogs, with an emphasis placed on supporting owners to effectively restrict food to maintain a healthy weight, acknowledging the challenges that owners are likely to face dealing with persistent food seeking behaviour.

### Dogs as a model of behavioural susceptibility theory

Additionally, dogs provide a relatable animal model that demonstrates how there is wide variation in food drive across individuals in a population, likely driven by genetic differences. The data shows that eating behaviours that predispose dogs to obesity render some individuals particularly susceptible to environmental exposure to an obesogenic environment that provides ready access to high calorie food and/or little physical activity.

We showed high food drive in dogs can overwhelm the good intentions of owners who try, but frequently fail, to limit their pets’ weight gain. This is relevant to humans because society frequently considers human obesity as the consequence of poor lifestyle choices and dismisses variation in food drive as a lack of self-regulation. In the canine model, the individual experiencing the food drive (dog) is separate from the person exerting control over food intake and activity (owner), compellingly illustrating the need for greater effort to maintain healthy weight in individuals with high food drive. Finally, the data shows how highly food motivated individuals are prone to weight gain unless considerable effort is made to prevent it, and that this contrasts with lower obesity risk in less food motivated individuals who are relatively resistant to an obesogenic environment.

## Conclusion

This work showed that high food motivation is a key driver of obesity in dogs, and that high food motivation renders affected dogs particularly susceptible to an obesogenic environment whereas dogs with low food motivation are relatively resistant to weight gain even where management might permit it. As well as being of veterinary interest, this is of relevance to human obesity as compelling, data-driven evidence of how behavioural susceptibility to environmental risk governs obesity outcome.

## Materials and methods

### Dog Obesity Risk Assessment (DORA) questionnaire

The Dog Obesity Risk Assessment (DORA) questionnaire [59], is a previously validated tool to assess dogs’ food motivation and owner management of diet and exercise, with high internal consistency (Chronach’s alpha > 0.8), short-term stability (Pearson’s correlation r > 0.8) and proven content, construct and criterion validity as well as external validity demonstrated by consistent results in later publications [14, 49, 74]. Collection of responses of the Dog Obesity Risk and Appetite (DORA) Questionnaire was approved by the Ethics and Welfare Committee of the Department of Veterinary Medicine, University of Cambridge (CR125).

Respondents were recruited through social media and local veterinary practices. In total, 19,885 responses to the DORA questionnaire and owner-reported Body Condition Score (BCS) were collected between 13th May 2015 and 17th October 2021 by Qualtrics software (Porto, UT). Respondents were not limited by geography but were predominantly from the United Kingdom, plus the USA, and other English-speaking countries where the social media ‘groups’ were accessed.

Dogs were included if: they were > 1 year old, because eating patterns might not be well established at younger ages; < 19 years old because dogs assigned higher ages were likely to be data entry error; and BCS > 3 because lower scores are clinically underweight and often associated with health problems. Breed-average values were only calculated for breeds with more than 10 dogs represented.

### DORA questionnaire scoring

To analyse the survey responses, we used Qualtrics Software (Porto, UT) and converted answers into a numerical format by following the scoring protocol described previously by Dr Eleanor Raffan [59]. Under this protocol, answers are scored as a percentage so that for questions with 4 options ‘definitely true’ scores 1, ‘mainly true’ 0.6667, ‘somewhat true’ 0.3333, and ‘not at all true’ 0. Similarly, for questions with 5 options ‘always’ scores 1, ‘often’ 0.75, ‘sometimes’ 0.50, ‘rarely’ 0.25, and ‘never’ 0. Some scores are to be reversed, and the scores are allocated in the opposite direction (e.g. ‘definitely true’ scores 0 and not at all true’ scores 1). Questions were then grouped into one of the following factors: ‘Dog Factor 1 (Responsiveness and Satiety)’, ‘Dog Factor 2 (Lack of Fussiness)’, ‘Dog Factor 3 (Interest in Food)’, ‘Restriction of Human Food Score’, ‘Owner Intervention Score’ and ‘Exercise Taken Score’. These factor scores were calculated as follows:

$$= (sum\ of\ question\ scores\ in\ that\ factor/ sum\ of\ maximum\ question\ scores\ for\ that\ factor)$$

Overall combined Dog Food Motivation Score and Owner Control Score were then calculated using the factor scores. Factor scores were multiplied by the number of questions contributing to that factor.

$$Owner\ Control\ Score= (Owner\ Intervention*4 + Restriction\ of\ Human\ Food*4 + Exercise\ Taken\ Score*5)/ 13$$

$$Food\ Motivation\ Score= (Responsiveness\ and\ Satiety*7 + Lack\ of\ Fussiness*3 + Interest\ in\ Food*3)/ 13$$

#### Quantification and statistical analysis

Data was analysed using R Software version 4.2.2 [57].

### Assessment of food motivation score and BCS population wide

Stepwise regression was performed, using the step() function from the basic stats package in R [57], to generate a minimal model for food motivation. Initially, the covariates included were sex, neuter status and age allowing for two-way interactions. Sex and neuter status were both coded in a binary manner (0 = Female, 1 = Male and 0 = entire, 1 = neutered) so that they could be included in the model. Next, this process was repeated with a model including breed and AIC and Multiple R-Squared were compared. The models compered were:

$$Food\ Motivation \sim sex*neuter\ status$$

$$Food\ Motivation \sim sex*neuter\ status + breed$$

To assess variables that explained variability of BCS the process described above was repeated with the following models being compared:

$$BCS \sim sex*neuter\ status$$

$$BCS \sim sex*neuter\ status + breed$$

$$BCS \sim sex*neuter\ status + Food\ Motivation$$

### Breed-averaged food motivation score

Stepwise regression was performed, using the step() function from the basic stats package in R, to generate a minimal model for food motivation. Initially, the covariates included were sex, neuter status and age allowing for two-way interactions. Sex and neuter status were both coded in a binary manner (0 = Female, 1 = Male and 0 = entire, 1 = neutered) so that they could be included in the model. The stepwise regression removed age from the model, giving the final minimal model:

$$\begin{aligned} Food\ motivation \sim &sex*neuter\ status + sex*age \\&+ neuter\ status*age \end{aligned}$$

To calculate an adjusted food motivation score, the effect sizes of sex, neuter status and their interaction on food motivation were extracted from the model for each breed. The following formula was then used to calculate the adjusted food motivation score for each individual (See Scripts).

$$\begin{aligned} Adjusted\ Food\ Motivation\,=\,&Individua{l}^{\prime}s\ FMS-\left(\left(sex\upbeta -\text{coefficient} * sex\ of\ individual\right)- mean\ sex\ for\ the\ individua{l}^{\prime}s\ breed\right)\\&- \left(\left(neuter\ status\upbeta -\text{coefficient} * neuter\ status\ of individual\right)- mean\ neuter\ status\ for\ the\ individua{l}{\prime}s\ breed\right)\\&- (\left(\upbeta -coefficient\ for\ the\ interaction\ between\ sex\ and\ neuter\ status * the\ interaction\ term\ of\ the\ individual\right)\\&- mean\ interaction\ for\ the\ individual^{\prime}s\ breed) \end{aligned}$$

To test whether the differences between the mean adjusted food motivation scores for each breed was significant, a one-way ANOVA test was performed.

### Banfield Pet Hospitals health records data extraction

Data from the electronic medical record database of Banfield Pet Hospitals, a large veterinary group in the United States, was extracted for preventative care visits (e.g. annual check-ups) between March 4th 2015 and Jan 31 st 2018, for neutered pure-bred dogs of 46 breeds aged between 1 and 15 years old. Breeds were chosen to be also present in the Food Motivation Score dataset, and to have at least 50 individuals on average across the different combinations of sex and age. Given these limitations, and after confirmation that point estimates were highly correlated whether entire dogs were included or not, only neutered dogs were included in the results presented here. For each visit, the following variables were extracted – dog ID, breed, sex, age, body condition score and weight. A single visit was then randomly selected per pet to help prevent correlated residuals.

### Body condition score

Different Body Condition Score systems exist with the 1–9 scale having been best validated [18, 19, 34, 45]. Therefore, for data we collected prospectively alongside the DORA questionnaire responses, we used the 9-point scale (Additional file 6). However, Banfield Pet Hospitals recorded BCS on a 5-point scale so for the breed-averaged obesity probability calculation we used that information. The 5-point scale scores dogs as 1 (thin), 2 (underweight), 3 (ideal), 4 (overweight) and 5 (obese). Since scores of 1 and 5 are relatively rarely used, they were merged with ‘underweight’ and ‘overweight’ respectively, to create a more stable model. The final dataset contained data from 1,149,591 dogs. The median number of dogs per breed was 24,991 (range 3,291 to 117,319).

### Breed overweight/obesity probability modelling

A multinomial model on Body Condition Score was achieved by running a set of binomial models against the same reference category (‘Ideal’), which were then merged. This procedure was necessary because the dataset was too large to run a multinomial model across all Body Condition Score categories at one time. The binomial models used Firth’s bias reduced logistic regression [25] in order to deal with occasional separation in the data (where some combination of predictors is entirely of one category). Confidence intervals were estimated using simulation. Each binomial model used terms ‘Breed’, ‘Year of Age’ and ‘Sex’.

Interactions between Breed and Sex, and between Breed and Year of Age, were also allowed. Predicted probabilities and 95% confidence intervals for these were then estimated for each breed, for the three body condition score categories (‘thin/underweight’, ‘ideal’ and ‘overweight/obese’). The estimated probabilities assumed an age distribution within each breed that matched that across the whole population, and a 50:50 ratio of males to females. This analysis used the R packages data.table (for data handling), mbest [56] (for the logistic regressions) and multcomp [28] (for the predictions).

### Breed overweight/obesity probability vs breed average food motivation

The Breed-Averaged Overweight/Obesity Risk data was plotted against the mean Breed-Averaged Food Motivation Score for each of the 46 pure breeds from which both Breed-Averaged Food Motivation Score and Overweight/Obesity Probability was available. As a linear relationship was observed, a Pearson’s correlation test and a linear regression analysis were performed (See scripts). Additionally, linear regression analysis was performed to test how much of the variation in Breed-Averaged Overweight/Obesity Risk could be accounted for by differences in average food motivation between breeds (See scripts).

### Owner management, BCS and food motivation

The mean between the three owner management factors was created and named ‘Owner Control Score’. Pearson’s Correlation of BCS and ‘Food Motivation Score’ with ‘Owner Control’, ‘Owner Intervention’, ‘Restriction of Human Food’ and ‘Exercise Scores’ were calculated. Given high level of significance the population was divided by ‘Food Motivation Score’ tertiles, a linear model for BCS was performed as follows:

$$\begin{aligned} BCS \sim (OI + RHF + EX) \,*\,& FMS\ tertile + sex*neuter\ status \\&+ age*neuter\ status + sex* age \end{aligned}$$

Minimal modelling was performed with Akaike information Criteria, followed by ANOVA test and Post-Hoc Pairwise t-test with Holm Correction.

## Supplementary Information

Additional file 1. Owners tend to underestimate BCS of their dogs. Bubble chart with regression shows vet-reported BCS (vet BCS) against owner-reported BCS (Owner BCS) in 618 dogs. Blue line represents the line of best fit with 95% confidence intervals shown in the shaded grey area. Red line represents a correlation of 1 where Owner BCS is equal to vet BCS. Dots correspond to datapoints, where bigger size represents greater number of subjects. Owner-reported BCS (mean ± sd) was 4.96 ± 0.8 while vet BCS was 5.27 ± 0.95. This cohort however had very few overweight animals, therefore under-scoring of BCS by the owners is likely underrepresented in comparison to a true population with higher adiposity variability.

Additional file 2. Body Condition Score was significantly positively correlated with Food Motivation Score besides being significantly negatively correlated with Owner Control Score and its sub scores. Pearson’s correlations matrix shown between BCS, Food Motivation Score and Owner Management Factors in the whole population. In each cell correlation (r) is shown with displayed with *p*-value in parenthesis.

Additional file 3. Variability in Obesity Probability between breeds can be explained by breed differences in food motivation but not in activity levels or management factors. Predicted average probabilities (0–1) of being overweight/obese (4–5/5), by breed, in ascending order is shown (red), which was obtained from electronic medical records of 1.1 million neutered dogs of 46 different breeds seen at Banfield Pet Hospitals between March 4th 2015 and Jan 31 st 2018. Breed-averaged Exercise Score (0–1; green), breed-averaged Food Motivation Score (0–1; blue) and breed-averaged Owner Control Score (0–1; yellow), obtained from over 14,000 responses to the previously validated DORA questionnaire [22], calculated with weighted effect of sex, age and neuter status for each breed. Dots show the mean value for each breed, alongside lines of best fit.

Additional file 4. Effect of owner management and other risk factors on BCS were different between contrasting obesity risk groups. Effect size β and significance level of biological and environmental factors on BCS between dogs in different Food Motivation Score tertile are shown. Values were extracted from a simple linear regression model that included sex, neuter status and age with two-way interactions and Owner Intervention Score, Restriction of Human Food Score and Exercise Score in two-way interaction terms with Food Motivation Score tertile. To obtain comparable effect β values the model was run three times, each with a different Food Motivation Score tertile group as a reference range, allowing us to obtain β values for the corresponding tertile. In each cell, β value is expressed with level of significance in parentheses. Age effects are expressed per year; Sex (0 = Female and 1 = Male); neutering status (0 = entire, 1 = neutered).

Additional file 5. Dogs in higher Food Motivation Score tertiles tend to have significantly higher BCS and are subject to significantly higher Owner Control Score and Owner Intervention Score but significantly lower Restriction of Human Food Score. No significant difference in Exercise Score exists between Food Motivation Score tertile groups. Box and Whisker Plot of raw data for BCS by Food Motivation tertile is shown (A), where midline is the median, box illustrates 1 st and 3rd quartile, lines show minimum and maximum value and dots represent outliers. Violin Plots of raw data by Food Motivation tertiles subgroups are shown for Food Motivation Score (B), Age (C), Owner Control Score (D), Owner Intervention Score (E), Restriction of Human Food Score (F) and Exercise Score (G)*.* Black dot and line represent mean and standard deviation; white dot represents median; p-values at the top show significance level between Food Motivation Score tertile groups and at the bottom is described mean ± standard deviation for each group. Dogs were excluded due to owner reported BCS < 4 or unreported (*n* = 3,616) and due to age < 1 or > 19 (n = 699), leaving 14,960 answers to analyse. There were 7,534 females (70% neutered) and 7,426 males (66% neutered). Two hundred and forty-four breeds registered at the British Kennel Club, “other pure breeds” and crossbreeds were included. Out of these, 70 breeds contained 10 or more individuals. Mean and standard deviation for age was 5.41 ± 3.32 years.

Additional file 6. Nine-point Body Condition Score system (WSAVA chart).

## Data Availability

All data and code used in the analysis are available on the Apollo repository (University of Cambridge, https://www.repository.cam.ac.uk/), 10.17863/CAM.101515.2.
