# Supplementary figures and images for: Behavioural susceptibility to environmental influences in obesity– evidence from a companion animal model
Source: BMC Vet Res. 2026 Jan 5;22:3. doi: 10.1186/s12917-025-04990-8 (PMC12772086; doi:10.1186/s12917-025-04990-8)

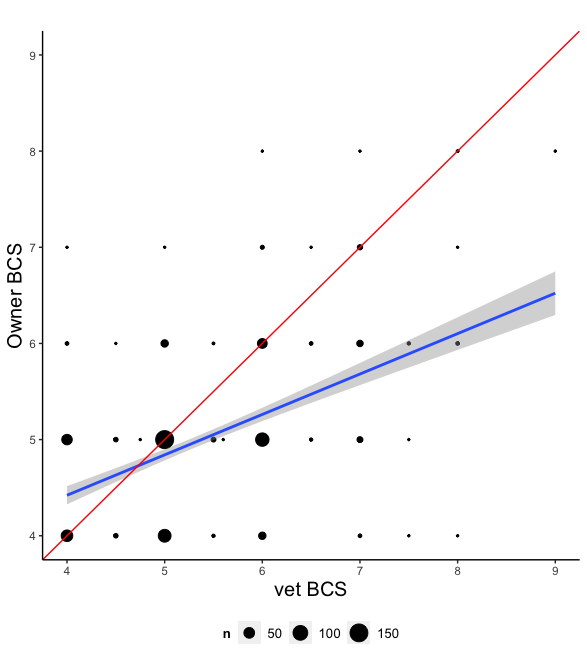

Supplement: Supplementary file 1 — Additional file 1. Owners tend to underestimate BCS of their dogs. Bubble chart with regression shows vet-reported BCS (vet BCS) against owner-reported BCS (Owner BCS) in 618 dogs. Blue line represents the line of best fit with 95% confidence intervals shown in the shaded grey area. Red line represents a correlation of 1 where Owner BCS is equal to vet BCS. Dots correspond to datapoints, where bigger size represents greater number of subjects. Owner-reported BCS (mean ± sd) was 4.96 ± 0.8 while vet BCS was 5.27 ± 0.95. This cohort however had very few overweight animals, therefore under-scoring of BCS by the owners is likely underrepresented in comparison to a true population with higher adiposity variability. [file 12917_2025_4990_MOESM1_ESM.png]

Breed average values

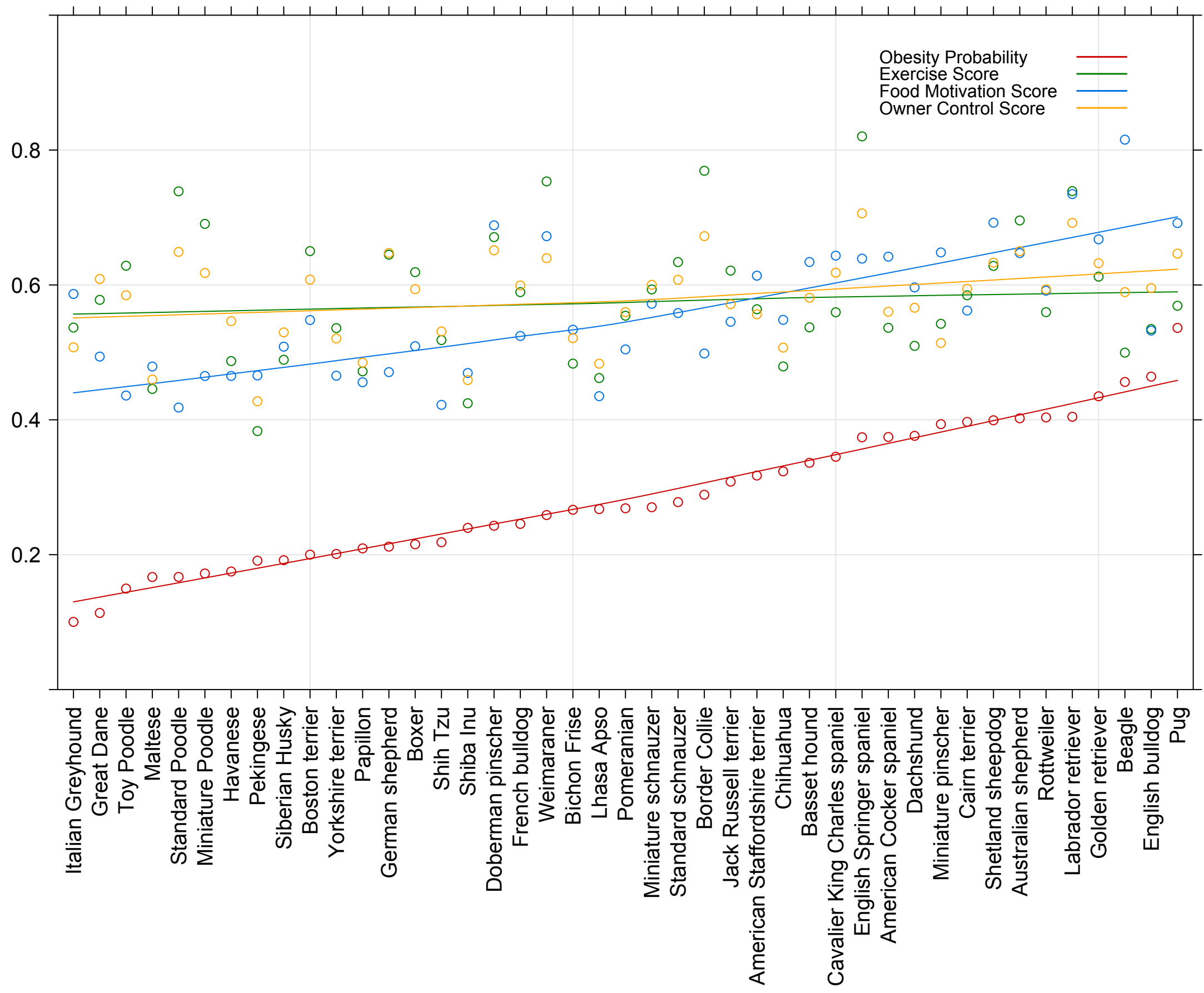

Supplement: Supplementary file 3 — Additional file 3. Variability in Obesity Probability between breeds can be explained by breed differences in food motivation but not in activity levels or management factors. Predicted average probabilities (0–1) of being overweight/obese (4–5/5), by breed, in ascending order is shown (red), which was obtained from electronic medical records of 1.1 million neutered dogs of 46 different breeds seen at Banfield Pet Hospitals between March 4th 2015 and Jan 31 st 2018. Breed-averaged Exercise Score (0–1; green), breed-averaged Food Motivation Score (0–1; blue) and breed-averaged Owner Control Score (0–1; yellow), obtained from over 14,000 responses to the previously validated DORA questionnaire [22], calculated with weighted effect of sex, age and neuter status for each breed. Dots show the mean value for each breed, alongside lines of best fit. [file 12917_2025_4990_MOESM3_ESM.pdf]

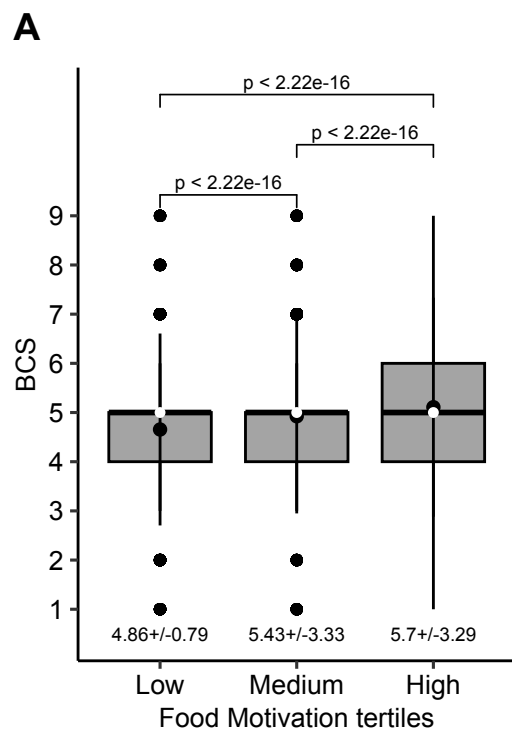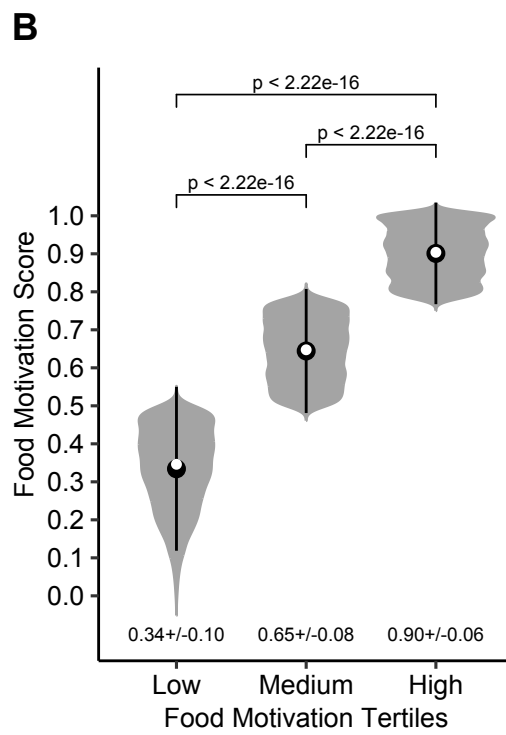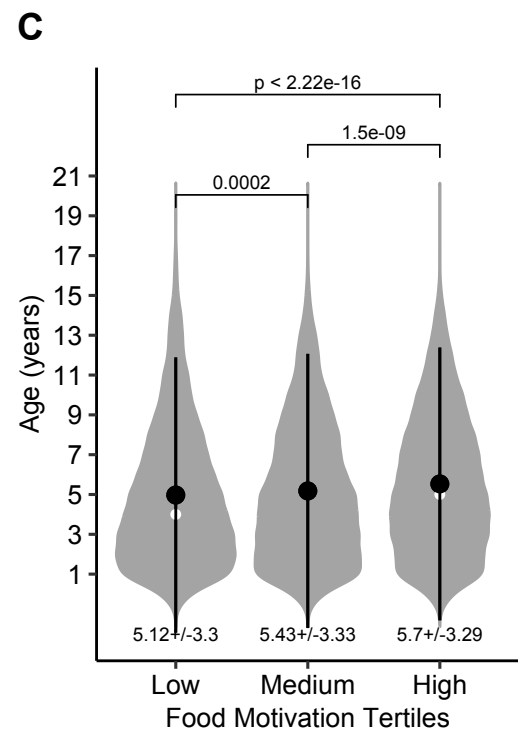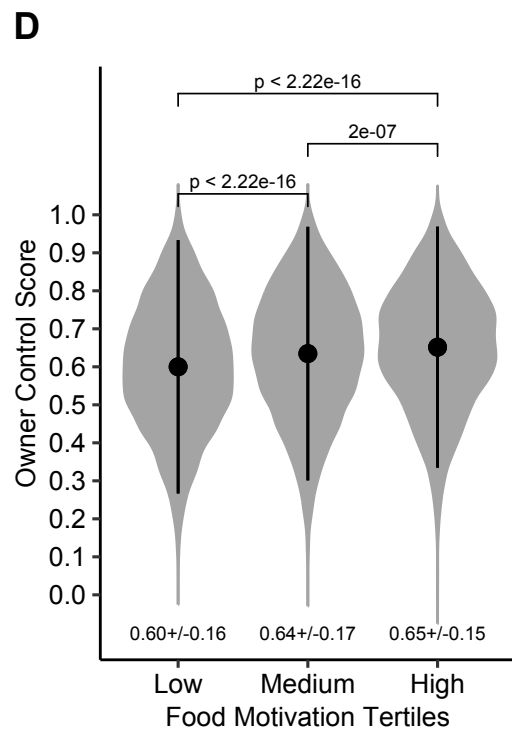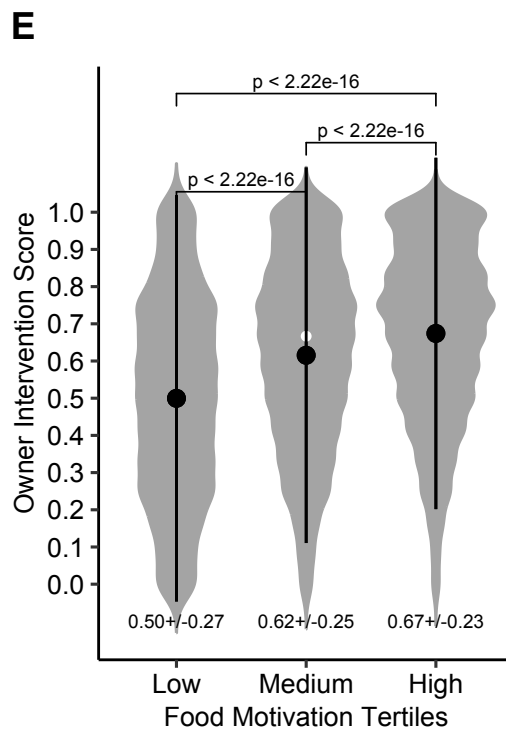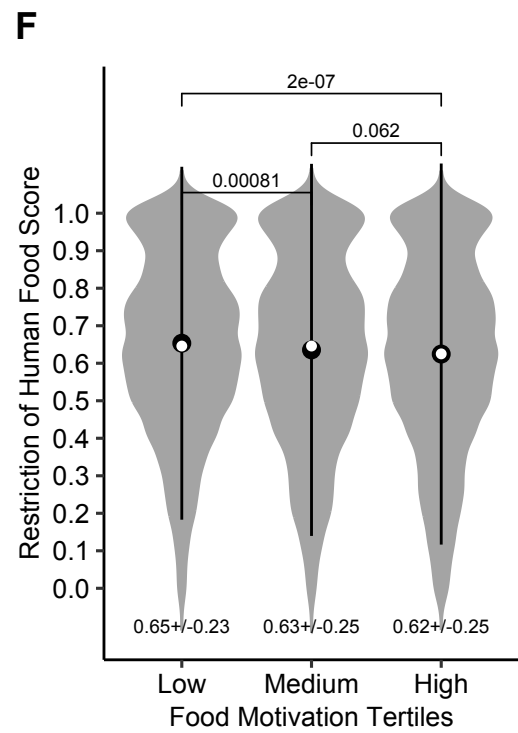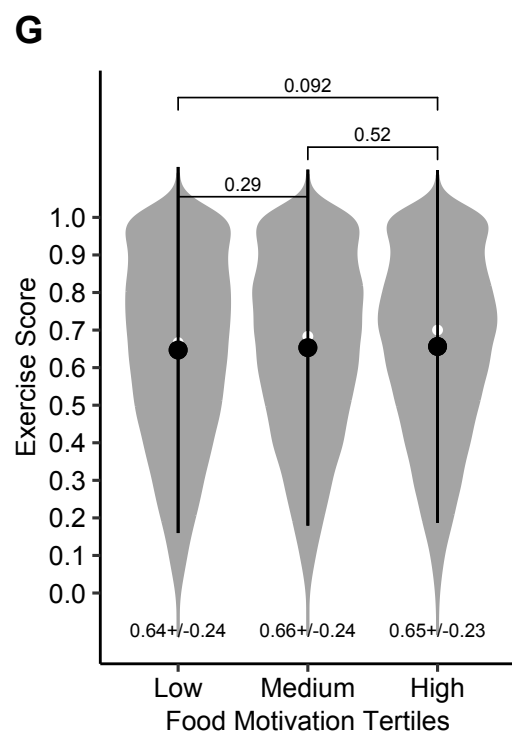

Supplement: Supplementary file 5 — Additional file 5. Dogs in higher Food Motivation Score tertiles tend to have significantly higher BCS and are subject to significantly higher Owner Control Score and Owner Intervention Score but significantly lower Restriction of Human Food Score. No significant difference in Exercise Score exists between Food Motivation Score tertile groups. Box and Whisker Plot of raw data for BCS by Food Motivation tertile is shown (A), where midline is the median, box illustrates 1 st and 3rd quartile, lines show minimum and maximum value and dots represent outliers. Violin Plots of raw data by Food Motivation tertiles subgroups are shown for Food Motivation Score (B), Age (C), Owner Control Score (D), Owner Intervention Score (E), Restriction of Human Food Score (F) and Exercise Score (G). Black dot and line represent mean and standard deviation; white dot represents median; p-values at the top show significance level between Food Motivation Score tertile groups and at the bottom is described mean ± standard deviation for each group. Dogs were excluded due to owner reported BCS < 4 or unreported (n = 3,616) and due to age < 1 or > 19 (n = 699), leaving 14,960 answers to analyse. There were 7,534 females (70% neutered) and 7,426 males (66% neutered). Two hundred and forty-four breeds registered at the British Kennel Club, “other pure breeds” and crossbreeds were included. Out of these, 70 breeds contained 10 or more individuals. Mean and standard deviation for age was 5.41 ± 3.32 years. [file 12917_2025_4990_MOESM5_ESM.pdf]
