# Supplementary material for: Behavioural susceptibility to environmental influences in obesity– evidence from a companion animal model
Source: BMC Vet Res. 2026 Jan 5;22:3. doi: 10.1186/s12917-025-04990-8 (PMC12772086; doi:10.1186/s12917-025-04990-8)
Supplement: Supplementary file 2 — Additional file 2. Body Condition Score was significantly positively correlated with Food Motivation Score besides being significantly negatively correlated with Owner Control Score and its sub scores. Pearson’s correlations matrix shown between BCS, Food Motivation Score and Owner Management Factors in the whole population. In each cell correlation (r) is shown with displayed with p-value in parenthesis. [file 12917_2025_4990_MOESM2_ESM.docx]

|  | **FMS** | **Owner Control** | **Owner Intervention** | **Restriction of Human Food** | **Exercise** | **Age** |
| --- | --- | --- | --- | --- | --- | --- |
| **BCS** | 0.19  (<0.001) | -0.22  (<0.001) | -0.06  (<0.001) | -0.15  (<0.001) | -0.23  (<0.001) | 0.18  (<0.001) |
| **FMS** |  | 0.12  (<0.001) | 0.29  (<0.001) | -0.07  (<0.001) | 0.01  (0.267) | 0.07  (<0.001) |
| **Owner Control** |  |  | 0.73  (<0.001) | 0.64  (<0.001) | 0.61  (<0.001) | -0.13  (<0.001) |
| **Owner Intervention** |  |  |  | 0.22  (<0.001) | 0.19  (<0.001) | 0.00  (0.973) |
| **Restriction of Human Food** |  |  |  |  | 0.06  (<0.001) | -0.07  (<0.001) |
