# Supplementary material for: Behavioural susceptibility to environmental influences in obesity– evidence from a companion animal model
Source: BMC Vet Res. 2026 Jan 5;22:3. doi: 10.1186/s12917-025-04990-8 (PMC12772086; doi:10.1186/s12917-025-04990-8)
Supplement: Supplementary file 4 — Additional file 4. Effect of owner management and other risk factors on BCS were different between contrasting obesity risk groups. Effect size β and significance level of biological and environmental factors on BCS between dogs in different Food Motivation Score tertile are shown. Values were extracted from a simple linear regression model that included sex, neuter status and age with two-way interactions and Owner Intervention Score, Restriction of Human Food Score and Exercise Score in two-way interaction terms with Food Motivation Score tertile. To obtain comparable effect β values the model was run three times, each with a different Food Motivation Score tertile group as a reference range, allowing us to obtain β values for the corresponding tertile. In each cell, β value is expressed with level of significance in parentheses. Age effects are expressed per year; Sex (0 = Female and 1 = Male); neutering status (0 = entire, 1 = neutered). [file 12917_2025_4990_MOESM4_ESM.docx]

|  | **Whole population** | **Low FMS** | **Medium FMS** | **High FMS** |
| --- | --- | --- | --- | --- |
| **Owner Intervention** | 0.33  (5.42X10^-6^) | 0.11  (0.016) | -0.22  (9.5x10^-6^) | -0.48  (<2x10^-16^) |
| **Restriction Human Food** | -0.17  (0.037) | -0.33  (3.59x10^-10^) | -0.42  (<2x10^-16^) | -0.47  (<2x10^-16^) |
| **Exercise** | -0.40  (6.25X10^-7^) | -0.60  (<2x10^-16^) | -0.76  (<2x10^-16^) | -0.85  (<2x10^-16^) |
| **Age** | 0.03  (1.68x10^-5^) | 0.03  (<2x10^-16^) | 0.03  (<2x10^-16^) | 0.03  (<2x10^-16^) |
| **Sex** | -0.09  (0.037) | -0.15  (4.66x10^-7^) | -0.18  (1.16x10^-8^) | -0.24  (3.42x10^-12^) |
| **Neuter status** | -0.05  (0.30) | 0.03  (0.263) | 0.07  (0.013) | 0.15  (4.x86x10^-6^) |
