# Supplementary material for: Behavioural susceptibility to environmental influences in obesity– evidence from a companion animal model
Source: BMC Vet Res. 2026 Jan 5;22:3. doi: 10.1186/s12917-025-04990-8 (PMC12772086; doi:10.1186/s12917-025-04990-8)
Supplement: Supplementary file 6 — Additional file 6. Nine-point Body Condition Score system (WSAVA chart). [file 12917_2025_4990_MOESM6_ESM.pdf]

WSAVA
Global Nutrition
Committee

Body Condition Score

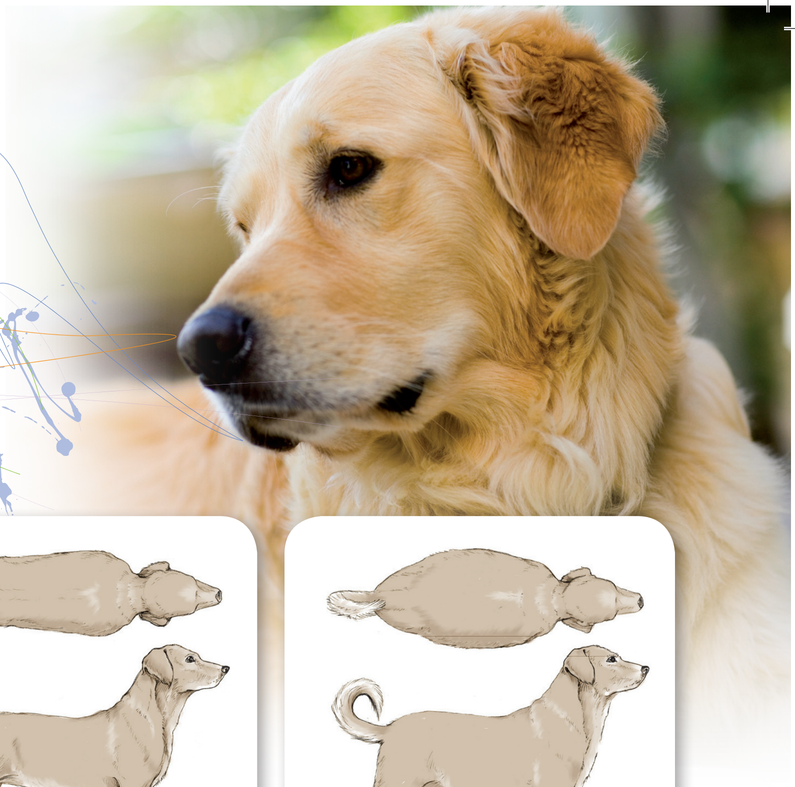

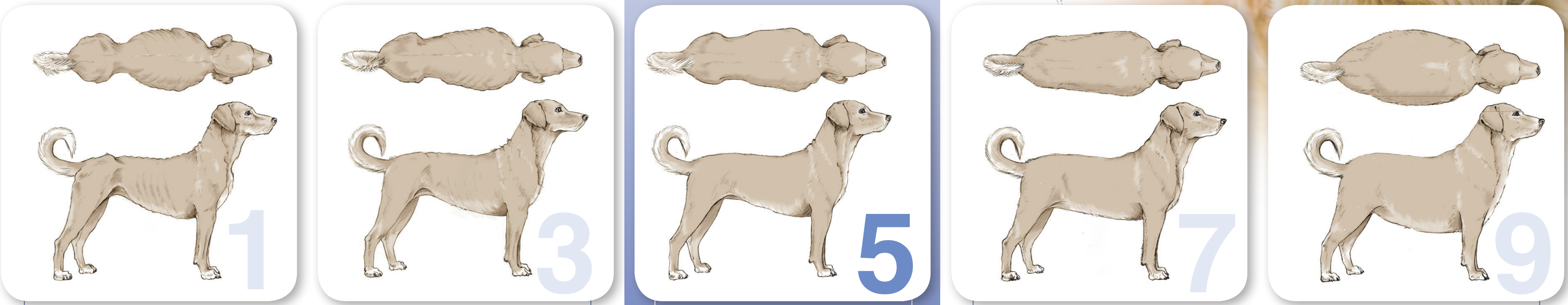

UNDER IDEAL

1. Ribs, lumbar vertebrae, pelvic bones and all bony prominences evident from a distance. No discernible body fat. Obvious loss of muscle mass.
2. Ribs, lumbar vertebrae and pelvic bones easily visible. No palpable fat. Some evidence of other bony prominences. Minimal loss of muscle mass.
3. Ribs easily palpated and may be visible with no palpable fat. Tops of lumbar vertebrae visible. Pelvic bones becoming prominent. Obvious waist and abdominal tuck.

IDEAL

4. Ribs easily palpable, with minimal fat covering. Waist easily noted, viewed from above. Abdominal tuck evident.
5. Ribs palpable without excess fat covering. Waist observed behind ribs when viewed from above. Abdomen tucked up when viewed from side.

OVER IDEAL

6. Ribs palpable with slight excess fat covering. Waist is discernible viewed from above but is not prominent. Abdominal tuck apparent.
7. Ribs palpable with difficulty; heavy fat cover. Noticeable fat deposits over lumbar area and base of tail. Waist absent or barely visible. Abdominal tuck may be present.
8. Ribs not palpable under very heavy fat cover, or palpable only with significant pressure. Heavy fat deposits over lumbar area and base of tail. Waist absent. No abdominal tuck. Obvious abdominal distention may be present.
9. Massive fat deposits over thorax, spine and base of tail. Waist and abdominal tuck absent. Fat deposits on neck and limbs. Obvious abdominal distention.

German A, et al. Comparison of a bioimpedance monitor with dual-energy x-ray absorptiometry for noninvasive estimation of percentage body fat in dogs. *AJVR* 2010;71:393-398.
Jeusette I, et al. Effect of breed on body composition and comparison between various methods to estimate body composition in dogs. *Res Vet Sci* 2010;88:227-232.
Kealy RD, et al. Effects of diet restriction on life span and age-related changes in dogs. *JAVMA* 2002;220:1315-1320.
Laflamme DP. Development and validation of a body condition score system for dogs. *Canine Pract* 1997;22:10-15.

©2013. All rights reserved.

wsava.org
